# The impact of alcohol care teams on emergency secondary care use following a diagnosis of alcoholic liver disease - a national cohort study

**DOI:** 10.1186/s12889-016-3350-0

**Published:** 2016-08-02

**Authors:** Claire Currie, Alisha Davies, Cono Ariti, Martin Bardsley

**Affiliations:** 1 Nuffield Trust, 59 New Cavendish St, London, W1G 7LP UK; 2 London School of Hygiene and Tropical Medicine, Keppel Street, London, W1CE 7HT UK

**Keywords:** Alcoholic liver disease, Hospital emergency service, Hospitalisation, Patient care team

## Abstract

**Background:**

The increasing mortality rates from alcohol-related liver disease (ARLD) are a public health concern. To address this, alcohol care teams (ACT) case-find and lead management of alcohol issues for these patients. Local assessments of ACTs have shown reductions in emergency admissions and emergency department attendances. We examine the impact of ACTs on emergency hospital activity following a diagnosis of ARLD.

**Methods:**

Administrative Hospital Episode Statistics (HES) data were extracted. Information on ACT provision at English NHS hospital trusts and sites in 2009/10 was taken from a survey by Public Health England. We undertook a difference-in-difference analysis to compare emergency hospital activity for a cohort of individuals diagnosed with ARLD who presented to hospitals either with or without an ACT in the one year before and after a first ARLD diagnosis during 2009/10.

**Results:**

Over the study period, 9,165 individuals eligible for inclusion in our study had a first diagnosis of ARLD. 4,768 presented to one of 41 hospital trusts with an ACT (59 sites) and 4,397 presented to one of 50 non-ACT hospital trusts (65 sites). Whilst age and sex demographics were similar between the two cohorts, the ACT hospital cohort had a higher proportion of individuals in the most deprived quintile (41.6 % v 28.5 % *p* < .0001). In the difference-in-difference analysis, the presence of an ACT at a hospital trust was not associated with a change in all-cause emergency admissions (0.020 (95 % CI −0.070, 0.111), *p* = 0.656), alcohol-related emergency admissions (−0.025 (95 % CI −0.104, 0.054), *p* = 0.536) or all-cause emergency department attendances (0.042 (95 % CI −0.087, 0.171), *p* = 0.521). Sensitivity analyses by sex and hospital site did not affect the study findings.

**Conclusions:**

In this study, the presence of an ACT at the NHS hospital trust where individuals have their first recorded diagnosis of ARLD does not appear to be associated with subsequent emergency hospital activity within these populations. Further analysis focussing on the components and specific effects of ACT interventions on individuals and systems both pre- and post-diagnosis of ARLD may reveal important avenues to improve care.

## Background

In the United Kingdom, standardised mortality rates from liver disease have increased 400 % since 1970, and in patients younger than 65 years have increased by almost 500 % (from 1.83 per 100,000 in 1970 to 9.46 per 100,000 in 2010) [1]. Consequently, liver disease is the third biggest cause of premature mortality with 62 000 years of working life lost every year [2]. In England and Wales 600,000 people have some form of liver disease of whom 60,000 people have cirrhosis, leading to 57,682 hospital admissions and 10,948 deaths in 2012 [3]. Nearly three-quarters of liver disease mortality is secondary to excess alcohol use [4] and rates of alcohol consumption remain a concern [1]. Timely intervention to reduce alcohol use has the capacity to prevent deterioration in liver disease [5]. A case-control study from the UK demonstrates that patients with alcoholic liver cirrhosis have an increased rate of alcohol-related admissions in the year prior to diagnosis compared to the general population and therefore there is an opportunity for preventative interventions in secondary care [6, 7].

According to Public Health England (PHE), 75 % of England’s hospitals in 2013 had an alcohol care team (ACT). Approach of these teams vary but roles may include facilitation of case-finding strategies, leading medical management of patients with alcohol problems, and liaising with community and secondary care services [8]. Local evaluations have shown alcohol teams to be effective in terms of reducing emergency admissions, reducing length of stay and improving quality of life across a range of patient groups [9–15]. A multi-disciplinary team at a hospital in Salford, UK, who targeted those with the highest number of alcohol related admissions saw a reduction in emergency department attendances and admissions when comparing the three months before and after the intervention period [11]. In a pragmatic randomised controlled trial, referral to an alcohol health worker at an inner London hospital was associated with a mean of 0 · 5 fewer visits to the emergency department over the following 12 months compared to the control arm of information alone [15]. However, to date, there have been no studies that have examined the impact of alcohol teams on hospital activity using national, administrative hospital data. The National Confidential Enquiry on Patient Outcome and Death (NCEPOD) identified opportunities to intervene earlier in alcohol-related liver disease (ARLD) to improve outcomes and we wanted to explore whether there was population-level evidence that ACTs were associated with beneficial health effects for ARLD patients.

Given the national policy emphasis on prevention [16] and potential challenges of funding preventative services as a result of fiscal constraints, we were keen to explore whether there was potential to identify population differences in emergency hospital activity associated with the presence of an ACT, using national, administrative data. Our hypothesis was that presence of ACTs at hospitals where a first recorded diagnosis of ARLD was made would be associated with a reduction in emergency hospital activity when examining the difference in activity before and after this point in comparison to where there were no ACTs.

## Methods

### Identifying ARLD patients

We defined a cohort of individuals from HES (the administrative dataset of activity in England’s NHS hospitals) using the unique pseudonymised identifier, aged 15 to 100 years who had a ‘first’ recorded diagnosis (using all diagnoses recorded) of ARLD (ICD-10 code K70) during 2009/10 in inpatient or outpatient HES (i.e. no recorded diagnosis of ARLD from April 2004 to March 2009). We defined the date of this admission or attendance as the index date for each patient.

### Assessing the potential impact of hospital alcohol teams

From the Public Health England (PHE) survey of hospital ACT provision carried out in 2013 [8], 114 services provided a date when the alcohol service has been set up (range from 2002 to 2013). We chose to define our cohort of patients in 2009/10 as approximately half of hospital ACTs have been set up since that time according to the PHE survey and offer a sufficient study population for analysis.

Using this information we identified hospital sites that had an alcohol service (ACT hospital) in the year of diagnosis of the ARLD cohort (2009/10), and those known not to have an alcohol team (non-ACT hospital) in 2009/10. We assigned ‘unknown’ status where no start date for the ACT had been reported in the PHE survey.

We linked this information about provision of hospital ACTs to the cohort of patients who had a first recorded diagnosis of ARLD in 2009/10. This was based on where patients had a potential exposure to a hospital ACT through there being a team in place in the hospital trust (the organisation which may be a group of hospitals over several locations, rather than the hospital site which is the hospital at a single location) where the patient presented to at the time of their first recorded diagnosis.

This information was used to examine whether provision of a hospital ACT had any impact upon counts of hospital activity, specifically emergency admissions and emergency department attendances for any reason and specifically alcohol related emergency admissions (Table 1) in the year following a first recorded diagnosis of ARLD compared to activity in the preceding year (based upon the index date for each person). Activity which occurred at any hospital was included as we considered hospital admission with a first recorded diagnosis to be a trigger for on-going individual support or behaviour change. This trigger event supported our choice of a one year before and after time period for comparison as well as this being informed by published local evaluations of effective services and in order to contain uncertainty in attribution of effect to ACT intervention.

**Table 1:** Wholly alcohol attributable ICD-10 codes used to define alcohol related inpatient activity (alcohol specific conditions)

ICD-10 code	Description
E24.4	Alcohol induced pseudo-Cushing’s syndrome
F10	Mental and behavioural disorders due to alcohol
G31.2	Degeneration of nervous system due to alcohol
G62.1	Alcoholic polyneuropathy
G72.1	Alcoholic myopathy
I42.6	Alcoholic cardiomyopathy
K29.2	Alcoholic gastritis
K70	Alcoholic liver disease
K85.2	Alcohol-induced acute pancreatitis
K86.0	Alcohol induced chronic pancreatitis
Q86.0	Fetal alcohol syndrome (dysmorphic)
R78.0	Excess alcohol blood levels
T51.0	Ethanol poisoning
T51.1	Methanol poisoning
T51.9	Toxic effect of alcohol, unspecified
X45	Accidental poisoning by and exposure to alcohol
X65	Intentional self-poisoning by and exposure to alcohol, undetermined intent
Y15	Poisoning by and exposure to alcohol, undetermined intent
Y90	Evidence of alcohol involvement determined by blood alcohol content
Y91	Evidence of alcohol involvement determined by level of intoxication

Source: Public Health England [27]

We carried out sub-group analyses by examining whether there were differences in the effect by sex. We also undertook a sensitivity analysis in which we only linked provision of a hospital ACT at a specific hospital site to the patients who had their first recorded diagnosis of ARLD at that hospital site.

### Statistical methods

A three level categorical variable was used to define the exposure of having the index admission at a trust with an ACT (has an ACT, no ACT and unknown). We described the patient characteristics in the ACT and no ACT groups, specifically, age, sex, ethnicity and deprivation measured by the proportion of the group in the most deprived 20 % of the English population (based on Index of Deprivation 2010 scores at lower super output area [17]) according to the patients place of residence and the proportion who died in hospital within the one year follow up. We examined whether there were important socio-demographic characteristics of the groups using chi-squared tests for proportions and a t-test for the difference in mean age.

We undertook a difference in difference analysis [18], using a linear mixed model [19], where we examined the difference in mean counts of hospital activity for the group of individuals presenting to hospitals with an ACT in the one year before diagnosis and compared this to those without for the same patient cohort in the subsequent year. This method was chosen to enable an assessment of the effect of the ACT intervention while overcoming problems of information bias (improved recording over time in A&E data is a recognised limitation of this dataset [20, 21] by using a comparator group, and confounding, by effectively matching patients to themselves in the year before and year after measures. The difference in difference analyses did not require further adjustment for confounding factors as this method takes account of time invariant confounding factors [22]. We would expect age, sex and deprivation over the two year time period examined to fall into this category. We included patient as a random effect to reflect the fact that the prior and subsequent hospital activity is correlated within a patient. All analysis was undertaken using SAS 9.4.

## Results

### Definition of the ARLD cohort

**Fig. 1:**
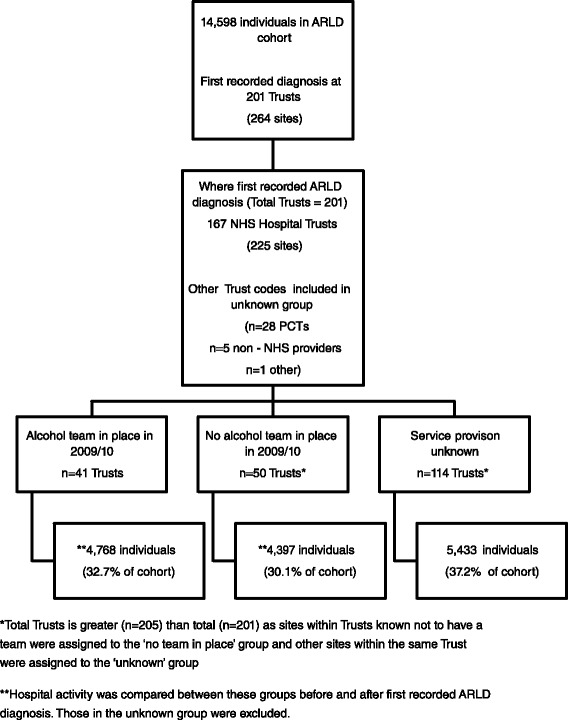
Number of cohort who had first recorded diagnosis at a hospital according to provision of an alcohol team

During 2009/2010, there were 27,450 individuals who had an admission with a primary or secondary diagnosis of ARLD on the HES dataset. From this group, 12,334 were excluded as they had previous ARLD activity (Fig. 1) recorded since April 2004. There were a further 518 exclusions because the patients was aged less than 15 years of age or was not resident in England. This left a cohort of 14,598 patients who had their first recorded diagnosis of ARLD during 2009/10.

### Definition of ACT service at hospital level

Responses to the PHE survey gathered start dates for 114 alcohol services [8]. Of these, 59 hospital sites, across 41 hospital trusts, had an ACT in place in 2009/10 or earlier and 65 sites, across 50 hospital trusts, were identified as not having an ACT service in 2009/10.

Of our 14,598 individuals diagnosed with ARLD in 2009/10, 4,768 individuals had their first recorded diagnosis at an ACT hospital, 4,397 at a non-ACT hospital and 5,433 in a hospital where the ACT service provision was unknown (Fig. 1). The unknown group were excluded from further analysis, leaving a cohort of 9,165 individuals with a first diagnosis of ARLD for analysis in 2009/10 linked to a hospital with known ACT provision.

### Comparison of the cohorts

The distributions of gender and age were similar between cohorts within ACT and non-ACT hospital trusts. There was no difference in one year in-hospital survival between the groups (Table 2). A greater proportion of individuals in the ACT hospital cohort were in the most deprived quintile (41.6 % v 28.5 % *p* < .0001) (Table 2). Ethnicity was unknown for the majority of individuals.

**Table 2:** Characteristics of ARLD cohort according to whether they had their first recorded diagnosis at an NHS hospital trust which had or did not have an alcohol team in 2009/10

Characteristic of ARLD cohort	Alcohol team		
	Yes (*n* = 4,768)	No (*n* = 4,397)	*p* value^a^
Male (%)	3290 (69.0 %)	3057 (69.5 %)	0.5878
Mean (SD) age (years)	53.6 (12.8)	54.2 (12.7)	0.0171
Age range (years)	17–94	18–95	
Resident in most deprived quintile of IMD (%)	1983 (41.6 %)	1253 (28.5 %)	<0.0001
White British ethnicity^b^ (%)	358 (7.5 %)	448 (10.2 %)	<0.0001
Died in hospital during one year follow up	1125 (23.6 %)	1020 (23.2 %)	0.6367

*SD* standard deviation

^a^Chi squared test for proportions (% male, % in most deprived Index of Multiple Deprivation (IMD) quintile, % White British & % one-year in-hospital mortality) and t-test for difference in mean age

^b^Ethnicity was unknown for the majority of individuals

We found small differences in the baseline activity in hospitals with and without ACTs (Table 3). In the year before diagnosis, the average number of (all cause and alcohol related) emergency admissions per patient was very similar in ACT and non-ACT hospitals, yet the average number of all-cause emergency department attendances appeared higher in the group where an ACT was present (Table 3).

**Table 3:** Difference-in-difference analysis on patients presenting to NHS hospital trusts with alcohol teams compared to NHS hospital trusts without - Estimated intervention effects for emergency hospital activity in the year before and the year after diagnosis (as mean per patient)

	Hospital trusts with an alcohol team (*n* = 4768)			Hospital trusts with no alcohol team (*n* = 4397)				
	One year before diagnosis (SD)	One year after diagnosis (SD)	Difference (SE)	One year before diagnosis (SD)	One year after diagnosis (SD)	Difference (SE)	Difference in difference (95 % CI)	*P*-value
Emergency admissions (all)	1.666 (1.69)	1.349 (2.31)	−0.318 (0.032)	1.691 (1.92)	1.353 (2.37)	−0.338 (0.033)	0.020 (−0.070, 0.111)	0.656
Emergency admissions (alcohol related)	1.301 (1.37)	1.074 (2.01)	−0.227 (0.028)	1.283 (1.49)	1.081 (2.04)	−0.202 (0.029)	−0.025 (−0.104, 0.054)	0.536
Emergency department attendances (all)	1.437 (3.08)	1.620 (4.72)	0.182 (0.046)	1.117 (2.43)	1.257 (3.31)	0.140 (0.047)	0.042 (−0.087, 0.171)	0.521

Data are mean number per patient (standard deviation) and difference (standard error). *P*-value is from the linear mixed model. *CI* confidence interval

Note: The ‘one year before diagnosis’ field includes the index admission which accounts for the mean number of emergency admissions being above one in this time period

### Impact of ACTs on emergency admissions and emergency department attendances

The difference-in-difference analysis comparing secondary care use between the two cohorts is shown in Table 3. While average emergency admissions, for any cause, reduced in the year after diagnosis for both the ACT and non-ACT hospital cohorts, there was no difference in the size of this reduction between the two groups. The reduction in the mean number of all-cause emergency admissions was 0.020 (95 % CI −0.070, 0.111), *p* = 0.656 smaller in the ACT group.

For alcohol related emergency admissions, there was also no significant difference in the observed reduction in mean number during the year after diagnosis between the ACT and non-ACT groups (−0.025 (95 % CI −0.104, 0.054), *p* = 0.536).

On average, patients had more all-cause emergency department attendances in the year following the first recorded diagnosis of ARLD in the ACT and non-ACT group. There was no significant difference in the observed increase in mean number during the year after diagnosis between the ACT and non-ACT groups (0.042 (95 % CI −0.087, 0.171), *p* = 0.521).

### Sensitivity analysis

In a sex-specific sub-analysis, there was no discernible difference in the findings between men and women for all-cause or alcohol related emergency admissions, or all-cause emergency department attendances (Appendices 1 and 2).

We carried out a sensitivity analysis in which we only linked provision of a hospital alcohol team at a specific hospital site to the patients who had their first recorded diagnosis of ARLD at that hospital site (Appendix 3). Of the 14,598 individuals as part of the incident ARLD cohort in 2009/10, 1,025 individuals in the cohort had their first recorded diagnosis at a hospital where an alcohol team was in place, 1,245 did not and 12,328 were unknown. The larger unknown group was due to incomplete use of site specific codes in the HES dataset.

There was no difference in the size of the reduction in mean number of emergency admissions (all cause) observed in the year following diagnosis compared to the year before, between the ACT and non-ACT groups (the reduction in mean number of all-cause emergency admissions was 0.103 (95 % CI −0.079 to 0.285), *p* = 0.267 fewer in the ACT group). For alcohol related emergency admissions, there was no difference in the observed reduction in the mean number during the year after diagnosis between the ACT and non-ACT groups (0.010 (95 % CI −0.150 to 0.169), *p* = 0.906) in hospital sites with an ACT. Finally, for all-cause emergency department attendances, in the year following the first recorded diagnosis of ARLD the increase from the year before was unaffected (0.031 (95 % CI −0.229 to 0.291), *p* = 0.816) by the presence on an ACT at the hospital site (Appendix 3).

## Discussion

### Main findings

This study was the first example of an analysis of the impact of ACTs on emergency hospital activity amongst an ARLD cohort at a national level using routine administrative datasets. We used the national PHE hospital alcohol team survey and English NHS hospital episode statistics to investigate the impact of ACTs on hospital activity for patients’ before and after diagnosis with ARLD. We proposed that presence of ACTs at hospitals where a first recorded diagnosis of ARLD was made would be associated with a reduction in emergency hospital activity when examining the difference in activity before and after this point in comparison to where there were no ACTs as a result of dedicated support to help these patients. However, using this approach where individuals in the ACT group may or may not have received the intervention, we did not find evidence of a change in emergency admissions (all cause or alcohol related) or all-cause emergency department attendances between hospitals with an ACT and those without a service.

Several other local evaluations of hospital alcohol services have shown ACT’s to be effective in reducing admissions, bed days, emergency department attendances and quality of care [9–14]. These evaluations have focused on a single service and were able to restrict their evaluations to individuals who had definitely received an ACT intervention. Furthermore, they often used a local database or audit data to capture hospital activity for the patients followed up over time. The generalisability of the findings of these single service studies are limited as ACTs deliver care often designed and applied to the local context. Our current study, by contrast, wanted to assess the effect of ACTs at a national level in order to provide support for commissioning of preventative services.

### Strengths and limitations

The cohort study design permitted the examination of ACT effects in a specific high need population. Our study was also more inclusive than previously published local evaluations in that it considered all secondary care activity within hospitals that allowed a comparative study to be undertaken at a national level and incorporated a robust statistical method to adjust for confounding factors.

It is important to consider this work in light of some limitations. We used the PHE survey of hospital ACT provision [8] to determine which individuals in our ARLD cohort could have potentially received an intervention. We therefore did not know which individuals had received an ACT intervention. This limited the power of the study as it is reasonable to assume that not every individual presenting to an ACT hospital would have received an intervention because of limited capacity or working hours. Defining our incident ARLD cohort in 2009/10 was useful in that there was a substantial group of hospitals at this time which did and which did not have an alcohol team in place. We attributed responsibility for supporting the individual to the hospital where the patient had their first recorded diagnosis. We acknowledge that coding practice may vary between hospitals and therefore may not be an exact measurement of the point of diagnosis. This is expected to be a source of random error in our comparison groups as coding practice is unlikely to be influenced by the presence of an ACT. Once the hospital admission or outpatient appointment has ended, the individual may rely on referral and follow up by services in the community for on-going support. We had no information on community or primary care service provision in this dataset. In addition, where the index admission was away from a patients usual place of residence, we had no information to ascertain any follow up arrangements and where links are not in place between services it is likely that this may have been difficult. This was not accounted for in our analyses and may have been a factor in examining the effect of potential intervention by an ACT given our focus on emergency hospital activity.

From the available data, we were only able to make a dichotomous assessment of ACT provision. The PHE survey had collected some information on the models of alcohol services provided in hospitals, however, it was difficult to categorise these services into specific types due to substantial variation in the operational models and due to incomplete information. This included, for instance, the number of whole time equivalent staff in the team, whether a weekend and evening service was provided, whether the team was led by a senior clinician, whether the service targeted patients in emergency departments or specific wards. It was also possible that services may have evolved over time and the information in the survey reflected the snapshot in time when the survey was completed.

For pragmatic reasons, we used 2009/10 as the timepoint to define when a hospital had an ACT. The retrospective nature of the PHE survey we used to identify ACT provision does not allow us to accurately identify if an ACT was active in a particular hospital over the whole 2009/10 period.

There are a variety of drivers of emergency care activity, particularly in patients with ARLD. The provision or cessation of health and community support services within individual localities may have affected the results of this study, but our datasets have not allowed us to take these into account. However, any impact is likely to have been random across ACT and non-ACT groups due to multiple factors influencing local community provision of a service such as competing needs, priorities and availability of funding.

We have also not considered if the patients have developed more complex ARLD over time. There are limited clinical variables in the HES dataset and this has precluded us from measuring severity of ARLD through the Model for End-stage Liver Disease [23] or Childs-Pugh [24] scores.

Additionally, for those hospitals that we included in our category as not having a dedicated alcohol team, it is plausible that frontline staff may have delivered brief interventions as part of their routine practice. Brief interventions have been shown to be effective [25] and so it is possible that this may have limited our ability to detect a difference between the hospitals which did and which did not have alcohol teams. It is also reasonable to assume that newly commissioned alcohol services would take some months to become fully effective as has previously been recognised [9] and this was not accounted for in the analysis. There may have been other initiatives which we have not been able to consider in this analysis.

We used first recorded inpatient admission or outpatient appointment with an ARLD diagnosis as a proxy for the date of ARLD diagnosis. We also anticipated that diagnosis would be a trigger for offering support to the patient. Ideally, harmful alcohol consumption would be picked up before this point, however, even if this is the case, the individuals included in the cohort went on to receiving a diagnosis of ARLD and so may have benefitted from further support at this point. We recognise that hospital alcohol teams are unlikely to only focus on patients who have a diagnosis of ARLD and the patients that we included in this analysis were therefore at the severe end of the spectrum of alcohol related harm.

### Explanation

There are several potential explanations for our findings. There may have been some important differences between the ACT hospital and non-ACT hospital cohorts. A larger proportion of individuals that had their first recorded ARLD diagnosis at an NHS hospital trust where an ACT was present were in the most deprived quintile of the English population and this may be an indication of greater need in this group. The higher all-cause emergency department attendances in ACT hospital trusts suggests these services were in areas with greater need. While our analyses accounted for time invariant confounders in the before and after measures (this would include deprivation over the two year period examined), it may have been that provision of an ACT avoided hospital activity that would otherwise have occurred in its absence. However, it is plausible that our measurement of exposure to an ACT was not suitably precise to detect the true effect of the intervention.

Interestingly, we observed an increase in average all-cause emergency department attendances per patient in the year following diagnosis regardless of whether the patient presented to an ACT or non-ACT hospital. We have already highlighted that improved recording of attendances in the A&E HES dataset over the time period we examined is a recognised limitation of these data (2,3). We also recognise there is local variation in coding practice which we would consider to be a source of random error in the measurement of A&E attendances at both the ‘year before’ and ‘year after’ time periods. However, it is also possible that this increase may reflect an increase in health seeking behaviour triggered by diagnosis of ARLD and lack of support or service provision in the community.

### Future work

To overcome some of these challenges in examining the impact of ACTs within NHS settings on acute care activity, more precise measurement of exposure to ACT’s is needed. This could be achieved through working with a subset of hospitals to obtain details of ACT provision and use this information to restrict the analyses accordingly. Along with greater certainty of ACT provision, it may also be possible to gather data of individuals who received an ACT intervention and link these, maintaining anonymity of individuals, to HES data.

Furthermore, to overcome challenges of potential differences in baseline need in ACT and non-ACT groups, an alternative method, such as a retrospective matched control method, could be used to generate a matched control group from the same hospital population as the cases receiving an ACT intervention (where individuals who have received the ACT intervention are known). This method could be applied to replicate and potentially validate findings of a previous local evaluation demonstrating an effective service.

In order to replicate the methodology of this study with more precise measurement of exposure to an ACT, greater granularity is required in hospital data. For instance, a well-designed dataset, with appropriate linkage, collecting patient level information on when individuals are referred to a hospital alcohol team (or all substance misuse services), what intervention was received, the individuals alcohol consumption status and relevant diagnoses would facilitate this and ideally should be joined up with community service provision. Alongside this, repeated surveys by PHE to monitor the changing picture of alcohol teams in England’s hospitals and would enable more in-depth examination of which aspects of hospital alcohol teams optimise their effectiveness.

Whilst the Five Year Forward View (describing how the NHS in England needs to change to meet the current challenges and changing demands, published by NHS England in 2014) [16] and the Government’s Alcohol Strategy 2012 (setting direction for alcohol policy in England and published under the Coalition government) [26] identify the importance of preventative services, constrained public health resources will drive the need to demonstrate higher quality evidence of efficacy. The increasing health and economic burden of liver disease [1] in the UK makes this a key priority for national preventative action.

## Conclusions

In our population level analyses the presence of an ACT at the NHS hospital trust where individuals had their first recorded diagnosis of ARLD did not appear to affect, subsequent emergency hospital activity. However this is likely to be due to a number of methodological challenges and a lack of patient level data on the ACT intervention. The evidence of increased liver-related mortality and the burden of alcohol on secondary care utilisation are important public health concerns. An increased focus on accurate reporting of the components and specific effects of ACT interventions on individuals and systems both pre- and post-diagnosis of ARLD will help build evidence of effectiveness and share learning across the country.

## Appendix 1

**Table 4:** Difference-in-difference analysis on male patients presenting to NHS hospital trusts with alcohol teams compared to NHS hospital trusts without - Estimated intervention effects for emergency hospital activity in the year before and the year after diagnosis (as mean per patient)

	Hospital sites with an alcohol team (*n* = 3,290)			Hospital sites with no alcohol team (*n* = 3,057)				
	One year before diagnosis (SD)	One year after diagnosis (SD)	Difference (SE)	One year before diagnosis (SD)	One year after diagnosis (SD)	Difference (SE)	Difference in difference (95 % CI)	*P*-value
Emergency admissions (all)	1.696 (1.800)	1.398 (2.412)	−0.298 (0.040)	1.717 (1.994)	1.401 (2.435)	−0.315 (0.042)	0.018 (−0.095 to 0.131)	0.758
Emergency admissions (alcohol related)	1.340 (1.490)	1.114 (2.096)	−0.226 (0.035)	1.316 (1.578)	1.129 (2.146)	−0.187 (0.037)	−0.039 (−0.139 to 0.060)	0.442
Emergency department attendances	1.516 (3.353)	1.763 (5.207)	0.247 (0.060)	1.144 (2.518)	1.343 (3.613)	0.199 (0.062)	0.048 (−0.120 to 0.217)	0.575

Data are mean number per patient (standard deviation) and difference (standard error). *P*-value is from the linear mixed model. *CI* confidence interval

Note: The ‘one year before diagnosis’ field includes the index admission which accounts for the mean number of emergency admissions being above one in this time period

## Appendix 2

**Table 5:** Difference-in-difference analysis on female patients presenting to NHS hospital trusts with alcohol teams compared to NHS hospital trusts without - Estimated intervention effects for emergency hospital activity in the year before and the year after diagnosis (as mean per patient)

	Hospital sites with an alcohol team (*n* = 1,478)			Hospital sites with no alcohol team (*n* = 1,340)				
	One year before diagnosis (SD)	One year after diagnosis (SD)	Difference (SE)	One year before diagnosis (SD)	One year after diagnosis (SD)	Difference (SE)	Difference in difference (95 % CI)	*P*-value
Emergency admissions (all)	1.600 (1.422)	1.237 (2.072)	−0.363 (0.051)	1.631 (1.750)	1.241 (2.205)	−0.390 (0.053)	0.028 (−0.116 to 0.172)	0.707
Emergency admissions (alcohol related)	1.214 (1.067)	0.984 (1.789)	−0.229 (0.044)	1.208 (1.250)	0.972 (1.782)	−0.237 (0.046)	0.007 (−0.118 to 0.133)	0.910
Emergency department attendances	1.263 (2.334)	1.301 (3.354)	0.038 (0.062)	1.054 (2.200)	1.060 (2.482)	0.006 (0.065)	0.032 (−0.145 to 0.209)	0.724

Data are mean number per patient (standard deviation) and difference (standard error). *P*-value is from the linear mixed model. *CI* confidence interval

Note: The ‘one year before diagnosis’ field includes the index admission which accounts for the mean number of emergency admissions being above one in this time period

## Appendix 3

**Table 6:** Difference-in-difference analysis on patients presenting to NHS hospital sites with alcohol teams compared to NHS hospital trusts without - Estimated intervention effects for emergency hospital activity in the year before and the year after diagnosis (as mean per patient)

	Hospital trusts with an alcohol team (*n* = 1025)			Hospital trusts with no alcohol team (*n* = 1245)				
	One year before diagnosis (SD)	One year after diagnosis (SD)	Difference (SE)	One year before diagnosis (SD)	One year after diagnosis (SD)	Difference (SE)	Difference in difference (95 % CI)	*P*-value
Emergency admissions (all)	1.628 (1.769)	1.375 (2.496)	−0.254 (0.069)	1.696 (1.945)	1.340 (2.390)	−0.357 (0.062)	0.103 (−0.079 to 0.285)	0.267
Emergency admissions (alcohol related)	1.270 (1.390)	1.057 (2.077)	−0.214 (0.060)	1.296 (1.538)	1.072 (2.157)	−0.223 (0.055)	0.010 (−0.150 to 0.169)	0.906
Emergency department attendances	1.693 (3.834)	1.736 (4.864)	0.043 (0.098)	1.589 (2.917)	1.601 (4.099)	0.012 (0.089)	0.031 (−0.229 to 0.291)	0.816

Data are mean number per patient (standard deviation) and difference (standard error). *P*-value is from the linear mixed model. *CI* confidence interval

Note: The ‘one year before diagnosis’ field includes the index admission which accounts for the mean number of emergency admissions being above one in this time period
